# Glecaprevir and Maraviroc are high-affinity inhibitors of SARS-CoV-2 main protease: possible implication in COVID-19 therapy

**DOI:** 10.1042/BSR20201256

**Published:** 2020-06-02

**Authors:** Anas Shamsi, Taj Mohammad, Saleha Anwar, Mohamed F. AlAjmi, Afzal Hussain, Md. Tabish Rehman, Asimul Islam, Md. Imtaiyaz Hassan

**Affiliations:** 1 Centre for Interdisciplinary Research in Basic Sciences, Jamia Millia Islamia, Jamia Nagar, New Delhi 110025, India; 2 Department of Pharmacognosy, College of Pharmacy, King Saud University, Riyadh 11451, KSA

**Keywords:** Coronavirus disease 2019, drug repurposing, FDA approved drugs, molecular docking, SARS-CoV-2, virtual screening

## Abstract

Due to the lack of efficient therapeutic options and clinical trial limitations, the FDA-approved drugs can be a good choice to handle Coronavirus disease (COVID-19). Many reports have enough evidence for the use of FDA-approved drugs which have inhibitory potential against target proteins of Severe Acute Respiratory Syndrome Coronavirus 2 (SARS-CoV-2). Here, we utilized a structure-based drug design approach to find possible drug candidates from the existing pool of FDA-approved drugs and checked their effectiveness against the SARS-CoV-2. We performed virtual screening of the FDA-approved drugs against the main protease (M^pro^) of SARS-CoV-2, an essential enzyme, and a potential drug target. Using well-defined computational methods, we identified Glecaprevir and Maraviroc (MVC) as the best inhibitors of SARS-CoV-2 M^pro^. Both drugs bind to the substrate-binding pocket of SARS-CoV-2 M^pro^ and form a significant number of non-covalent interactions. Glecaprevir and MVC bind to the conserved residues of substrate-binding pocket of SARS-CoV-2 M^pro^. This work provides sufficient evidence for the use of Glecaprevir and MVC for the therapeutic management of COVID-19 after experimental validation and clinical manifestations.

## Introduction

Coronavirus disease (COVID-19) is a public health emergency across the globe that emerged from Wuhan, China in 2019. The causative agent of COVID-19 is a newly modified form of SARS coronavirus (SARS-CoV) that has increased pathogenicity and spread, known as Severe Acute Respiratory Syndrome Coronavirus 2 (SARS-CoV-2) [1]. Owing to the global impact, the World Health Organization (WHO) announced COVID-19 as a pandemic disease [2]. According to WHO as of 29 May 2020, this pandemic disease has 5,858,627 confirmed cases of infection with 359,994 confirmed deaths across the globe (https://www.worldometers.info/coronavirus/) since the first patient was hospitalized on 12 December 2019 [3], thus highlighting the menace caused by this deadly disease. Most of the infections are self-limited, with only approximately 15% of infected adults developing severe pneumonia that requires treatment with supplemental oxygen and an additional 5% progress to critical illness with hypoxemic respiratory failure, acute respiratory distress syndrome, and multiorgan failure that requires ventilatory support, often for several weeks [4,5].

Coronaviruses (COVs) are a group of extremely diverse, enveloped, positive-sense, and single-stranded RNA viruses [6], which cause a broad spectrum of respiratory, enteric, hepatic, and neurological diseases with varying severity among humans and animals [7]. Approximately six COV species were known to cause human diseases [8]. Out of these six, four viruses namely: 229E, OC43, NL63, and HKU1 are prevalent and characteristically cause common cold symptoms in the immunocompetent individuals [8]. Severe acute respiratory syndrome coronavirus (SARS-CoV) and Middle East respiratory syndrome coronavirus (MERS-CoV) were the other two, which were far more pathogenic than others thereby causing fatal illness. SARS-CoV‐2 is the seventh COV that can infect human and belongs to the β-COV group, and is extremely infectious, causing human‐to‐human transmission.

The spectrum of clinical manifestation of COVID-19 ranges from asymptomatic to severe respiratory failure [9]. The main symptoms comprise self-reported fever, fatigue, dry cough, myalgia, and dyspnea. In most of the SARS-CoV-2 cases, pneumonia is present but there are cases of pleuritic chest pain that have also been reported [4]. Based on symptoms severity, patients can be classified into mild, severe, and critical types [10].

The genomic features of SARS-CoV‐2 are well-matched with the family of coronavirus, but it has significant differences in the gene sequence as compared with previously sequenced CoVs [11]. Sequence comparison suggests approximately 79.5 and 96% sequence similarity of SARS-CoV-2 to the SARS-CoV and bat coronavirus, respectively. Despite a high sequence similarity, the difference is highly impactful which makes the SARS-CoV-2 resistant to the drugs used against SARS-CoV.

The envelope spike (S) protein is important for infection and pathogenesis of coronavirus [12]. SARS-CoV-2 uses S-protein is a trimeric class I viral fusion glycoprotein undergoes a structural rearrangement during fusion to the host cell membrane [13]. The angiotensin-converting enzyme II (ACE2) is known as a receptor for SARS-CoV2 [14]. The S-protein reacts with the ACE2 of the alveolar epithelial cells (pneumocytes) with a 10–20-fold higher affinity of SARS-CoV-2 in comparison with the S protein of SARS-CoV [15]. All these evidences made S protein a target for the development of a vaccine against COVID19.

Due to high sequence conservation and critical role in pathogenesis, the main protease (M^pro^, 3CL^pro^) is considered as an effective target for drug design and development [16]. The main function of SARS-CoV-2 M^pro^ is the regulation of replication and transcription, making it an attractive drug target for structure-based drug design and discovery [17,18]. M^pro^ cleaves polyproteins to generate non-structural proteins (NSPs) that form a replicase–transcriptase complex. There is high sequence similarity (96%) in SARS-CoV M^pro^ and SARS-CoV-2 M^pro^, recommended as an attractive drug target in COVID19 therapeutics [16]. SARS-CoV-2 M^pro^ consists of 306 amino acid residues. Crystal structure analysis suggests that Thr^25^, Thr^26^, Leu^27^, His^41^, Ser^46^, Met^49^, Tyr^54^, Phe^140^, Leu^141^, Asn^142^, Gly^143^, Cys^145^, His^163^, Met^165^, Glu^166^, Leu^167^, Pro^168^, Phe^185^, Asp^187^, Gln^189^, Thr^190^, Ala^191^, and Gln^192^ are found in the active site pocket of SARS-CoV-2 M^pro^. During drug development strategy these residues serve as a platform for the development of potent and selective inhibitors of SARS-CoV-2 M^pro^ [16].

Many antiviral drugs have been employed for the treatment of SARS-CoV-2 infection [6,19]. Five FDA-approved drugs (ribavirin, penciclovir, nitazoxanide, nafamostat, and chloroquine (CQ)) and two broad-spectrum antiviral drugs remdesivir (GS-5734) and favipiravir (T-705) were tested against the clinical isolate of SARS-CoV-2 [20]. Among these, remdesivir (GS-5734), an experimental drug developed for the treatment of Ebola virus and CQ, a malarial drug are showing considerable effectiveness [20,21]. Remdesivir is an adenosine analog, which incorporates into nascent viral RNA chains and results in premature termination [22]. CQ is a potential broad-spectrum antiviral drug that blocks viral infection by increasing endosomal pH mandatory for virus/cell fusion and interfering with glycosylation of cellular receptors of the virus.

However, an overdose of CQ can cause acute poisoning and death [23]. The clinical safety profile of hydroxychloroquine is better than CQ (during long-term use) in terms of treatment of SARS-CoV-2 infection but still has a fewer concerns [24]. As per recent reports, remdesivir is gaining attention as one of the most promising COVID-19 drug. Remdesivir has inhibitory effects on pathogenic animal and human coronaviruses, including SARS-CoV-2 *in vitro*, and inhibits MERS-CoV and SARS-CoV-1, and SARS-CoV-2 replication in animal [25]. Thus, remdesivir is considered as promissing drug in the clinical management of COVID-19 patients. Intravenous administration of remdesivir was adequately tolerated but did not provide significant clinical or antiviral effects in seriously ill COVID-19 patients [26], thus further studies are required to establish it as a successful player in COVID-19 therapeutics.

The repurposing of approved drugs provides an alternative approach to develop safe and effective therapeutics against rapidly emerging diseases such as COVID-19 [27]. FDA-approved C–C chemokine receptor type 5 (CCR5) receptor antagonist maraviroc (MVC) is effective to inhibit R5-tropic HIV-1 entry into cells [28]. MVC has a good pharmacokinetic profile, with relatively low protein binding and high bioavailability making it a good drug. Besides its role in HIV-1 infection, clinical trial data and animal studies suggest the protective role of MVC in different diseases including cancer [29], graft versus host, and inflammatory diseases [30]. In a recent study, MVC is recommended as a potential drug candidate to fight with COVID-19 [31]. Glecaprevir is another antiviral drug that acts on Hepatitis C virus (HCV) NS3/4A protease inhibitor thereby targeting the viral RNA replication [32]. Glecaprevir in combination with pibrentasvir has proven to be a highly effective pan-genotypic treatment for HCV patients without cirrhosis and with compensated cirrhosis, with a high barrier to resistance, and is safe and effective in patients with advanced renal disease, HIV, and solid organ transplants [33].

The timely development of effective and safe drugs for clinical purposes is very challenging as conventional drug development process usually takes years and costs billions [34]. Here we employed structure-based rational drug design to find potential inhibitors of SARS-CoV-2 M^pro^ to meet the immediate need of COVID-19 therapeutics. Structure-based virtual screening of the FDA-approved drugs was performed in search of high-affinity inhibitors of SARS-CoV-2 M^pro^. First, we estimated the binding affinities of the drugs with SARS-CoV-2 M^pro^, and then interaction analysis was carried out to find better hits. Finally, based on the interaction analysis and drug properties, we identified two drugs bearing appreciable binding affinity and specific interaction toward the substrate-binding pocket of SARS-CoV-2 M^pro^.

## Materials and methods

### Structure-based virtual screening

Various bioinformatics software, such as MGL Tools [35], AutoDock Vina [36], and Discovery Studio [37] were used for structure-based virtual screening. Several online resources such as the National Center for Biotechnology Information [38], RCSB Protein Data Bank (PDB) [39], and DrugBank [40] were used in retrieval, evaluation, and analysis of the data. The crystal structure of SARS-CoV-2 M^pro^ in complex with different inhibitors provide a basis to identify potential inhibitors [41]. We have extensively analyzed the available crystal structures of SARS-CoV-2 M^pro^ including the PDB IDs 5R7Y, 5R7Z, 5R80, 5R81, 5R83, 5R84, 5RE4, 5RE9, 5REB, 5RF1, 5EFQ, 6LU7, and 6W63 and investigated its mechanism of inhibition followed by the identification of critical residues of the binding pocket. For molecular docking-based virtual screening, we have taken the atomic coordinates of SARS-CoV-2 M^pro^ from the PDB (ID: 6M03). This PDB file was chosen for molecular docking-based virtual screening study because its apo form and higher resolution, thus no conformational changes were induced to its native structure by any co-crystallized ligand. Energy minimization using Swiss-PDB Viewer was performed on the protein structure to get the stable and lowest energy conformation state of SARS-CoV-2 M^pro^. The total internal energy (bonds, torsions, angles, improper, non-bonded, and electrostatic) of SARS-CoV-2 M^pro^ computed *in vacuo* with the GROMOS96 43B1 force-field was estimated as −12751.65 and −17940.15 kJ/mol before and after energy minimization, respectively.

The structure was prepared by adding hydrogen atoms to the polar groups in the protein along with the Kollman charges. The molecular docking was performed using AutoDock Vina where the screening was structurally blind for all the drugs with a grid size of 34, 62, and 62 Å, centralized at 12.11, −11.38, and 4.66 for X, Y, and Z coordinates, respectively. The grid spacing was set to 1.00 Å with the exhaustiveness of 8. The docking results were screened for high binding affinity and then all possible docked conformations were generated for each drug which was further analyzed using PyMOL and Discovery Studio for their possible interaction toward SARS-CoV-2 M^pro^. In PyMOL, the polar interactions formed within 3.5 Å were mapped and labeled as close contacts between the drugs and protein. The charged potential was also generated to the protein surface to explore the ligands binding in the deep cavity of SARS-CoV-2 M^pro^. Discovery Studio was employed to explore detailed interactions and their types including hydrogen bonds, halogen, alkyl, and the van der Waals interactions formed between the selected drugs and the protein, SARS-CoV-2 M^pro^. From the interaction analysis, only those drugs were selected which specifically interact to the active-site residues of SARS-CoV-2 M^pro^.

The p*K*i, the negative decimal logarithm of inhibition constant was calculated from the ∆*G* parameter while using the following formula-

(1) $$\Delta G=RT(\text{Ln}K{\text{i}}_{\text{pred}})$$

(2) $$K_{\text{i}}=e^{\left(\Delta G/RT\right)}$$

(3) $$pK_{i}(\text{μM})=-log(K{\text{i}}_{\text{pred}})\;\times 10^{-6}$$

where ∆*G* is binding affinity (kcal/mol), *R* (gas constant) is 1.98 cal*(mol*K)^−1^, and *T* (room temperature) is 298.15 Kelvin.

## Results and discussion

### Virtual screening

Virtual screening results in the identification of many drugs having appreciable docking scores with the SARS-CoV-2 M^pro^. Initially, we have selected the top 10 hits out of 2388 FDA-approved drugs showing a significant binding affinity to the SARS-CoV-2 M^pro^ in the range of −10.3 to −9.1 kcal/mol (Table 1). From the identified hits, we noticed that these drugs are implicated in a wide range of therapeutics including viral and neurological disorders.

**Table 1:** List of FDA-approved drugs showing remarkable binding affinity to the SARS-CoV-2 M^pro^

S.No.	Drug	Affinity (kcal/mol)	p*K*i_pred_ (µM)	Therapeutics
1.	Glecaprevir	−9.6	7.06	Protease inhibitor against chronic hepatitis C
2.	MVC	−9.4	6.91	HIV infection
3.	Nystatin	−9.3	6.84	Antifungal
4.	Dihydroergotamine	−10.3	7.58	Migraines
5.	Ergoloid	−9.4	6.91	Dementia and age-related cognitive impairment
6.	Lurasidone	−9.3	6.84	Schizophrenia and bipolar disorder
7.	Naldemedine	−9.9	7.28	Opioid-induced constipation
8.	Aprepitant	−9.3	6.84	Postoperative nausea and vomiting
9.	Eptifibatide	−9.1	6.69	Myocardial infarction
10.	Alisertib	−9.2	6.77	Refractory peripheral T-cell lymphoma

Abbreviations: p*K*i, negative decimal logarithm of inhibition constant; pred, predicted.

Detailed interaction analysis of all the docked conformers of top 10 hits was carried out to find specific interactions toward the SARS-CoV-2 M^pro^ binding pocket. Here, based on the interaction and drug-like properties, we identified two drugs, Glecaprevir and MVC which form many close interactions to a set of critically important residues of SARS-CoV-2 M^pro^. It has been observed that residues of SARS-CoV-2 M^pro^ such as Thr^25^, Thr^26^, Leu^27^, His^41^, Thr^45^, Ser^46^, Met^49^, Phe^140^, Leu^141^, Asn^142^, Gly^143^, Ser^144^, Cys^145^, His^163^, His^164^, Met^165^, Glu^166^, Leu^167^, Pro^168^, and Gln^189^ are offering a significant number of interactions to both the drugs. Both drugs are mimicking the same binding pattern where the co-crystallized inhibitors of SARS-CoV-2 M^pro^ bind.

**Figure 1:**
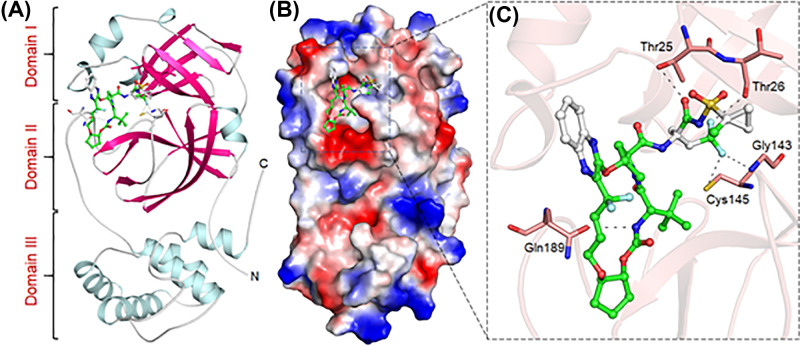
Binding pattern of Glecaprevir with SARS-CoV-2 M^pro^ (**A**) Structural representation of SARS-CoV-2 M^pro^ complexed with Glecaprevir. (**B**) Glecaprevir blocking the binding pocket. (**C**) Making significant interactions with the functionally important residues of SARS-CoV-2 M^pro^.

Both Glecaprevir and MVC are antiviral drugs used in the treatment of chronic hepatitis C and HIV infection, respectively. Glecaprevir is a direct-acting antiviral agent and HCV NS3/4A protease inhibitor that targets the RNA replication in viruses [42]. The binding pattern of Glecaprevir is indicating a strong fit to the binding pocket of SARS-CoV-2 M^pro^ which may hinder the substrate accessibility and subsequent inhibition (Figure 1). Glecaprevir binds in between the domain I and II where the substrate-binding site is located (Figure 1A). Glecaprevir is blocking the pocket (Figure 1B) and making significant interactions with critically important residues of SARS-CoV-2 M^pro^ (Figure 1C). These properties of Glecaprevir can make it a potential lead to developing SARS-CoV-2 M^pro^ inhibitors to be used as effective clinical molecules to combat against COVID-19.

**Figure 2:**
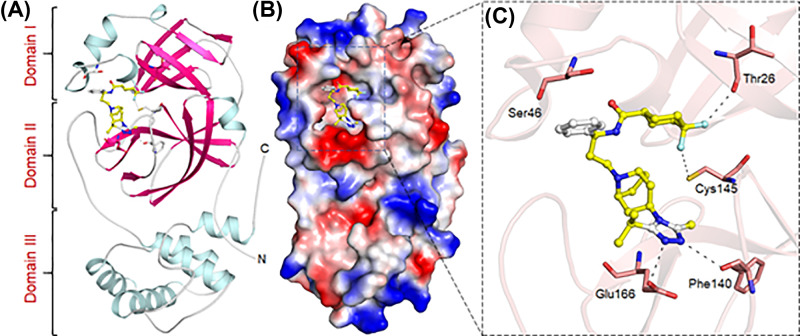
Binding pattern of MVC with SARS-CoV-2 M^pro^ (**A**) Structural representation of SARS-CoV-2 M^pro^ complexed with MVC. (**B**) MVC blocking the binding pocket, and (**C**) making significant interactions with the functionally important residues of SARS-CoV-2 M^pro^.

On the other hand, MVC is also an antiretroviral agent that inhibits the interaction between HIV and CCR5. MVC is an entry inhibitor that restricts the binding, fusion, and entry of an HIV virion to the human cell. By blocking this step in HIV’s replication, MVC slows the progression of HIV infection [43]. These properties make MVC a possible lead to test in COVID-19 infection. The binding pattern of MVC has suggested a strong binding to the pocket of SARS-CoV-2 M^pro^ which could result in strong inhibition of SARS-CoV-2 M^pro^ and blocking of COVID-19 infection (Figure 2). MVC also binds in between the domain I and II where the substrate-binding site is located and forms a significant number of interactions to the critically important residues of SARS-CoV-2 M^pro^.

**Figure 3:**
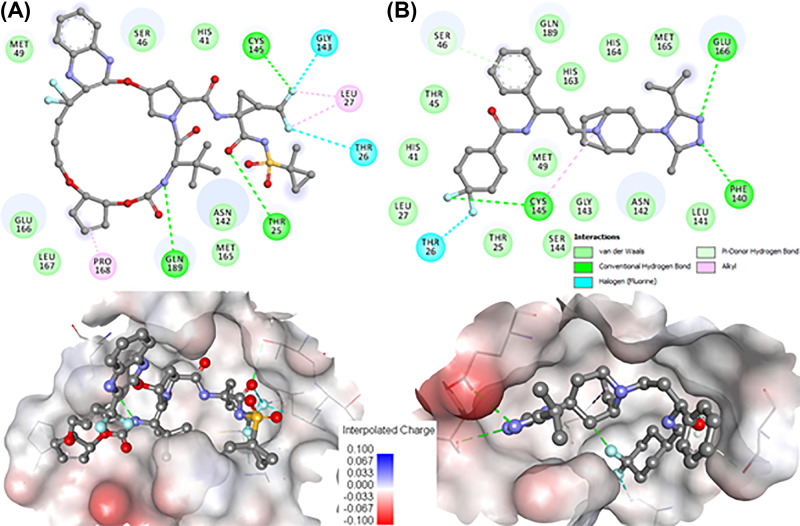
Detailed binding of Glecaprevir and Maraviroc with SARS-CoV-2 M^pro^ 2D plots of the SARS-CoV-2 M^pro^ binding-pocket residues and their interactions with (**A**) Glecaprevir and (**B**) Maraviroc. Lower panels are showing the surface representation of conserved substrate-binding pocket of SARS-CoV-2 M^pro^ complex with Glecaprevir and MVC, respectively (left to right).

Both Glecaprevir and MVC are docked to the binding pocket of SARS-CoV-2 M^pro^ were analyzed for their detailed interaction with the functionally important residues. It is evident from Figure 3 that both drugs are showing a significant number of interactions with the functionally important residues of the SARS-CoV-2 M^pro^ binding pocket. Glecaprevir is showing major interactions with the SARS-CoV-2 M^pro^ including three hydrogen bonds among Thr^25^, Cys^145^, and Gln^189^, and two fluorine interactions with Thr^26^ and Gly^143^ (Figure 3A, upper panel). Similarly, MVC forms many interactions with the SARS-CoV-2 M^pro^ including four hydrogen bonds with Ser^46^, Phe^140^, Cys^145^, and Glu^166^, and one fluorine interactions with Thr^26^ (Figure 3B, upper panel). Besides, both drugs fit well into the deep cavity of the M^pro^ binding pocket to hinder the substrate accessibility thus inhibition of enzyme activity. Both drugs share a similar binding pattern and many common interactions, along with the mimicking of the same orientation where most of the co-crystallized inhibitors of SARS-CoV-2 M^pro^ bind [41,44].

During interaction analysis, we found that both Glecaprevir and MVC form many close interactions including halogen and hydrogen bonds to the residues of the substrate-binding pocket, which helps to lock the inhibitor inside the substrate-binding pocket and thus effectively inhibit the SARS-CoV-2 M^pro^. The analysis suggested that the binding and therapeutic properties of Glecaprevir and MVC make them prominent leads to develop potential inhibitors of SARS-CoV-2 M^pro^. The chemical information of Glecaprevir and MVC is listed in Table 2 which might be further exploited to design potential and specific therapeutic molecules to combat COVID-19.

**Table 2:** List of the selected drugs and their chemical structures

Drug	IUPAC name	Molecular formula	Structure
Glecaprevir	(1R,14E,18R,22R,26S,29S)-26-tert-butyl-N-[(1R,2R)-2-(difluoromethyl)-1-[(1-methylcyclopropyl)sulfonylcarbamoyl]cyclopropyl]-13,13-difluoro-24,27-dioxo-2,17,23-trioxa-4,11,25,28-tetrazapentacyclo[26.2.1.03,12.05,10.018,22]hentriaconta-3,5,7,9,11,14-hexaene-29-carboxamide	C_38_H_46_F_4_N_6_O_9_S	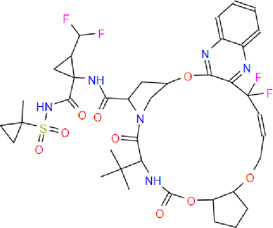
MVC	4,4-difluoro-N-[(1S)-3-[(1S,5R)-3-(3-methyl-5-propan-2-yl-1,2,4-triazol-4-yl)-8-azabicyclo[3.2.1]octan-8-yl]-1-phenylpropyl]cyclohexane-1-carboxamide	C_29_H_41_F_2_N_5_O	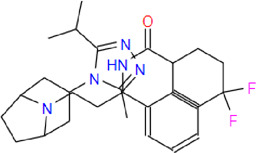

The ligand-bound structures of SARS-CoV-2 M^pro^ provide useful information for antiviral drug design against COVID-19 [45,46]. Taking the structural information of SARS-CoV-2 M^pro^ and its binding with several inhibitors, we identified two drugs Glecaprevir and MVC repurposing potential therapeutics against COVID-19.

## Conclusion

In the present study, we analyzed the structures of SARS-CoV-2 M^pro^ co-crystallized with different inhibitors and explored their binding pattern and mechanism of inhibition. Interaction analysis of the co-crystallized inhibitors binding to the substrate-binding site provides structural insights into the design of substrate-based inhibitors targeting SARS-CoV-2 M^pro^. Finally, we have identified two drugs, Glecaprevir and MVC binding to the substrate-binding pocket of SARS-CoV-2 M^pro^ which is highly conserved among all the structures of SARS-CoV-2 M^pro^. This strongly supports our hypothesis that the development of an antiviral molecule targeting SARS-CoV-2 M^pro^ or in combination with other potential strategies might be useful to develop an effective treatment of COVID-19. Inhibition of SARS-CoV-2 M^pro^ by FDA-approved drugs is an attractive therapeutic choice to handle the emergent need of COVID-19. However, experimental validation and clinical manifestation are required. The *in-silico* approach presented here can significantly contribute toward the quick discovery and development of potential drugs to manage rapidly emerging COVID-19.

## Abbreviations

ACE2: angiotensin-converting enzyme II
CCR5: C–C chemokine receptor type 5
COV: coronavirus
COVID-19: coronavirus disease
CQ: chloroquine
HCV: Hepatitis C virus
MERS-CoV: Middle East respiratory syndrome coronavirus
M^pro^: main protease
MVC: maraviroc
PDB: Protein Data Bank
SARS-CoV-2: severe acute respiratory syndrome coronavirus 2
WHO: World Health Organization
